# Scar tissue: after the pandemic

**DOI:** 10.1186/s44158-022-00078-z

**Published:** 2022-12-19

**Authors:** Nathan D. Nielsen

**Affiliations:** Division of Pulmonary, Critical Care and Sleep Medicine & Section of Transfusion Medicine and Therapeutic Pathology, University of New Mexico School of Medicine, Albuquerque, NM USA

**Keywords:** Pandemic, Burnout, Secondary traumatic stress, Compassion fatigue, Moral injury, Resilience

“Scar tissue that I wish you saw…

With the birds I’ll share this lonely viewin’.

With the birds I’ll share this lonely viewin’”.

         - *Scar Tissue*, Red Hot Chili Peppers

I have scars.

Every now and then when I look at my arms and legs, I notice them. Scars left behind from an overactive childhood, scars from my late teens as a competitive hurdler, scars from reconstructive knee surgery in my late 20 s, and scars from other misadventures in my 30 s. Most are faded to the point of a smooth silvery whiteness, though the post-veterinarian visit scratch from my cat last week remains angrily reddish-pink, edges still jagged. Tracing the edges of these scars transports me back to the moments in which they were first acquired, and events even now decades past become vividly clear. As the 1990s pop song goes, “Scars are souvenirs you never lose.”^1^ My scars will be with me always, inextricably bound to memory.

I have newer scars, though these are not visible to the eye. As with so many of us in healthcare, the two-plus years of the COVID pandemic left me with psychological injuries, a host of unwanted memories, and emotional baggage I confess I have yet to unpack. And I know of colleagues and friends who suffered far worse than I. As we find ourselves in this odd in-between time when COVID cases and deaths are blessedly rare but there has yet to be (and may never be) an official declaration of victory over the virus, the temptation of letting the terrible experiences of the pandemic slide into the realm of the forgotten is easy to succumb to. But there is danger in this easy forgetting—anyone at all familiar with the psychological sequelae of trauma knows too well the risks of suppressing or repressing traumatic experiences. Injuries poorly cared for leave the deepest and ugliest of scars. This is why it is so important to acknowledge that we as COVID-era healthcare practitioners were deeply injured by the events of the pandemic.

But where to begin? While achieving a full understanding of the long-term effects of the pandemic on our collective psyche will no doubt take years, in order to even *begin* to understand the nature of these injuries, we must first name their source: loss and hurt.

In the past 2 ½ years, all of us experienced loss of one sort or another. For some, the loss was tangible—I lost family, and my wife lost family. So many of us lost colleagues, friends, and family. As millions across the globe mourned the deaths of those they loved, healthcare workers were not spared this mourning. Far from it. And the grief of our personal losses often magnified the sense of powerlessness that so many of us felt as we struggled against the virus in our professional lives. Furthermore, the disruption of the traditional rituals of mourning and farewell only made the losses feel deeper, justified as we knew those disruptions to be.

Not only did we lose friends, family, and loved ones, but many of us also lost our faith.

We lost faith in our fellow man and woman, the “general public.” Those that fought against measures we knew would save lives—resisting masking, refusing vaccines, and spreading uninformed or intentional misinformation. Those who made ungrounded, angry demands for dangerous, ineffective treatments like hydroxychloroquine and ivermectin. And more recently, those directing escalating abuse, threats, and violence towards healthcare workers. How is it not possible to feel betrayed when the public that serenaded us with pots and pans one year turned around and threatened us with violence the next? Being pulled down from pedestals we never asked to be put on was an awkwardly unpleasant experience.

We lost faith in the media—for their politically slanted reporting, sensationalism, and minimization of the brutal truths of the pandemic that we had seen with our own eyes and for peddling unsubstantiated conspiracy theories and giving legitimacy to dangerous ideas that never deserved an iota of it. Voices that could have promoted life-saving behavior and encouraged a collective responsibility for the welfare of others were instead used to sow division and breed conflict in service of clicks and ratings.

(Of little surprise) we lost what limited faith we had in government—for their delayed and incoherent responses, for their denials of the truth, and for their distortion of facts for political gain. Time and again, we saw that when political leaders had unprecedented opportunities to create a unity of purpose, they instead turned them into occasions of self-promotion or the demonization of those with differing ideologies.

We lost faith in previously trusted institutions and organizations like the CDC and the WHO. As mentioned in the *New York Times* even this past week, “During Covid, officials have sometimes given unclear or misleading guidance because they did not trust the public with the truth.”^2^ Is it any surprise that we who had to deal day after day with the morbid and mortal consequences of this “unclear or misleading guidance” now view these institutions with suspicion? Even previously sacrosanct, incontrovertible “science” came into doubt as treatment guidances lurched from one pole to another.

And if you are anything like me, in the darkest days, we even lost faith in ourselves. I doubted my competence, my tools, and my training as I time and again failed to save those under my care. So little of what I did seemed to make a difference stemming the tides of suffering and loss that self-doubt crept into every decision I made in the ICU.

The injuries inflicted upon us by these manifold losses will take a significant amount of time to define, let alone come to terms with, far be it to recover from. In the nearer term, the other root cause of our collective distress is easier to identify—hurt.

Hurt is a strangely uncomfortable topic for those of us in the healthcare profession and recently has all too often been simplistically reduced to “burnout.” Burnout, commonly defined as “chronic workplace stress not managed successfully,” is nowhere near enough to describe our damage from the pandemic. To adequately describe the psychological hurt COVID caused us, we need to employ the language of *trauma*—for would any critical care practitioner not describe their pandemic experience as traumatic?

For the majority of us, though we will (thankfully) never meet the criteria for classic PTSD, many other elements of trauma psychology are still applicable. Among them is the concept of secondary traumatic stress (STS, sometimes called bystander or vicarious trauma). This refers to the emotional duress that results when an individual witnesses the traumatic experiences of another. STS can affect any healthcare provider at any time and has many similarities to PTSD, including hypervigilance, avoidance, flashbacks, and mood changes. It can also involve feelings of guilt and anger, cause problems with sleep and concentration, and lead to exhaustion. There are still rooms in my ICU that I avoid, as being near them floods me with horrible memories to this day—they are triggers for my STS, if you will.

Another relevant element is compassion fatigue. This is described as the emotional and physical exhaustion that leads to a diminished ability to empathize or feel compassion for others. Some have described it as the “cost of caring.” I certainly suffered from some of this—particularly in the post-vaccine waves of the pandemic, when so much suffering was so easily preventable.

One of the most painful elements of the pandemic experience, though, was repeated and severe moral injury. Moral injury represents what happens when clinicians have to make choices that violate our commitments and our principles. For me, not being able to accept the transfer of a 22-year-old young man with acute but very treatable leukemia to my hospital because my ICU was full of unvaccinated, science-denying COVID patients was a terrible moral injury. These moral injuries have consequences—strong feelings of shame, guilt, self-condemnation, and even a shattered (or at least bruised) sense of self.

The pandemic circumstances that led to moral injury will no doubt sound familiar to all of you reading this: rationing of scarce resources and terrible triage decisions that had to be made quickly and with incomplete and limited information, when decisions made “above our heads” presented us with impossible choices. And fear. The fear of being infected ourselves, of infecting those we love—knowing that our daily professional responsibilities put those we cared about at risk—that too caused moral injury.

We all experienced secondary trauma, compassion fatigue, and moral injury to different extents and in different ways due to COVID, and while we ourselves may lack the tools to process them without guidance, being able to describe these feelings and reactions as we go through our daily professional lives or as we look back upon our pandemic memories is an essential step towards coming to terms with them and beginning the healing process.

As I look at my own scars, physical and psychological, I am struck that scars are not intrinsically bad things. Scars are by definition a form of closure—marking a place where yes, damage has occurred, but also a place where there has been *healing*. They are a testament to survival—reminders of trauma endured and overcome. Whether scars remain ugly, reflecting irreversible damage to the tissues beneath, or with time fade to the point of near invisibility, depends on how they are tended to.

We can mourn those whom we have lost, but better yet, we can celebrate their lives. We can restore our lost faith in institutions by seeking out those truly worthy of our trust, but better yet, we can work to build them. We can find the faith we lost in our fellow men and women by communing with those who supported us during the dark pandemic days, but better yet, we can fight to forgive those that did not. We can choose to use the trauma, the fatigue, and the moral injuries we suffered as fuel for change—to change our hospitals, our profession, and our communities for the better.

While I cannot certainly provide the single prescription for healing the losses and hurts that the pandemic left behind, I can offer the following recommendations (though in the spirit of honesty, I must confess that I struggle to practice them):

- Return to the things that bring you joy.
- Write down your grief, your anger, your confusion. Even if nobody will ever read it but you, write anyway.
- Be kind to yourself.
- Look out for each other—remember that little acts of support, kindness, and caring can mean more than you know.
- Celebrate the friends and colleagues that propped you up during the difficult moments—and tell them how grateful you are for them.
- Honor the memory of the patients you lost, but equally so those that you saved—because of your efforts, there were families that did not have to mourn.
- Be honest with yourself, and if you are struggling, seek help. Our profession is notoriously poor at “healing ourselves”—this is a practice that needs to end.

Our COVID scars do not have to define us as people or as professionals, but they will remain with us in one form or another regardless. Whether they serve as reminders of our strength or as tethers to our pain depends on how we care for them *now*.

## Data Availability

Not applicable.

## Footnotes

1 *Name*, The Goo Goo Dolls, released 1998.

2 The New York Times, *The Morning*, October 13, 2022.

